# Validation of the implementation of phased-array heating systems in Plan2Heat

**DOI:** 10.1007/s00066-024-02264-0

**Published:** 2024-08-14

**Authors:** H. P. Kok, J Crezee

**Affiliations:** 1 Amsterdam UMC, University of Amsterdam, Dept. Radiation Oncology, Cancer Center Amsterdam, Meibergdreef 9, 1105 AZ Amsterdam, The Netherlands; 2 Cancer Center Amsterdam, Treatment and quality of life, Cancer biology and immunology, Amsterdam, The Netherlands

**Keywords:** Electromagnetic heating, Modeling, Plan2Heat, Validation, Locoregional hyperthermia

## Abstract

**Background:**

Hyperthermia treatment planning can be supportive to ensure treatment quality, provided reliable prediction of the heating characteristics (i.e., focus size and effects of phase-amplitude and frequency steering) of the device concerned is possible. This study validates the predictions made by the treatment planning system Plan2Heat for various clinically used phased-array systems.

**Methods:**

The evaluated heating systems were AMC-2, AMC-4/ALBA-4D (Med-Logix srl, Rome, Italy), BSD Sigma-30, and Sigma-60 (Pyrexar Medical, Salt Lake City, UT, USA). Plan2Heat was used for specific absorption rate (SAR) simulations in phantoms representing measurement set-ups reported in the literature. SAR profiles from published measurement data based on E‑field or temperature rise were used to compare the device-specific heating characteristics predicted by Plan2Heat.

**Results:**

Plan2Heat is able to predict the correct location and size of the SAR focus, as determined by phase-amplitude settings and operating frequency. Measured effects of phase-amplitude steering on focus shifts (i.e., local SAR minima or maxima) were also correctly reflected in treatment planning predictions. Deviations between measurements and simulations were typically < 10–20%, which is within the range of experimental uncertainty for such phased-array measurements.

**Conclusion:**

Plan2Heat is capable of adequately predicting the heating characteristics of the AMC‑2, AMC-4/ALBA-4D, BSD Sigma-30, and Sigma-60 phased-array systems routinely used in clinical hyperthermia.

**Supplementary Information:**

The online version of this article (10.1007/s00066-024-02264-0) contains supplementary material, which is available to authorized users.

## Introduction

The effectiveness of the radiotherapy and chemotherapy schemes applied for pelvic tumors (e.g., cervical, rectal, or bladder) can be significantly enhanced by adding moderate locoregional hyperthermia at 40–43 °C for 1 h once or twice a week [1]. Locoregional heating is often applied using radiative phased-array systems operating in the frequency range between 60 and 130 MHz [2, 3]. Phased-array systems used in clinical hyperthermia centers consist of waveguides or dipole antennas positioned in one or multiple rings around the patient. Commercially available phased-array systems are the BSD-2000 systems (Pyrexar Medical, Salt Lake City, UT, USA) [4, 5] and the ALBA-4D system (Med-Logix srl, Rome, Italy) [6]. The relatively large wavelength (λ), ranging between ~30 and 60 cm at the operating frequencies used, ensures good penetration of the electromagnetic energy for heating of deep-seated tumors. A focal zone at the location of the tumor is generated by phase-amplitude steering, inducing constructive interference between the electromagnetic fields emitted by the antennas. The large wavelengths also imply a relatively large heating focus of roughly ¼ λ, yielding targeted but also non-conformal and non-selective tumor heating, also including some temperature increase in surrounding normal tissues. This normal tissue heating is not an issue, because moderate hyperthermia yields tumor-selective radiosensitization effects [7].

Moderate normal tissue heating is well tolerated, but excessive normal tissue temperatures beyond the pain sensation threshold (hotspots) should be avoided as these can lead to thermal toxicity. Such high temperature rises typically occur at tissue interfaces with large inhomogeneities in dielectric tissue properties and perfusion, i.e., fat/muscle/bone. These hotspots can be power limiting and thus result in suboptimal tumor heating. Realizing sufficiently high tumor temperatures is important to ensure treatment quality, since a clear thermal dose–effect relationship exists [8–15]. The main challenge in clinical locoregional heating is therefore to realize the highest possible temperatures in the target region while avoiding treatment-limiting hotspots elsewhere. To monitor treatment quality, real-time thermometry feedback during treatment is essential and prescribed by quality assurance (QA) guidelines [16]. This thermometry is typically limited to minimally invasive probes in the natural body cavities (vagina, bladder, rectum) near the target region. In case of treatment-limiting hotspots, the patient experiences and reports a pain sensation (at ~45 °C) [17]. The system settings then need to be adjusted to modify the interference patterns such that a local temperature decrease at the hotspot location is realized while maintaining therapeutic tumor temperature levels in the target region.

Adequate phase-amplitude steering is thus an important aspect of locoregional hyperthermia treatments. The clinically used phased-array devices differ in terms of operating frequency, type of antenna, and antenna configuration. QA measurements are used to characterize heating devices, showing the basic characteristics such as focusing ability, focus size, and effects of phase-amplitude steering on the heating focus and validating system performance [16, 18]. This is done using a homogeneous tissue-equivalent phantom or a simplified inhomogeneous phantom, because these basic characteristics and variations in performance become less pronounced and more difficult to validate in more complex geometries or anatomies. QA measurements aim to characterize the performance of a hyperthermia device by determining specific absorption rate (SAR) patterns, which can be either based on electric field (E-field) or temperature rise (∆T) measurements, typically along one, two, or three main axes of the phantom [19]. E‑field measurements are performed in a liquid phantom using a scanning E‑field probe. The SAR relates to the measured E‑field as

1 $$SAR=\frac{\sigma }{2\rho }\left|\left|E\right|\right|^{2},$$

where *E* (V/m) is the electric field and σ (S/m) and ρ (kg/m^3^) are the conductivity and the density of the phantom material, respectively. Continuous scanning thus yields a high measurement resolution and a continuous measured profile. Temperature rise measurements on the other hand use a solid phantom with a limited number of thermometry probes at relevant locations. The temperature rise after a short power pulse (typically 1–6 min) is proportional to the SAR, since heat conduction effects are negligible over this short heating period. Conduction effects should be avoided since these will have a “blurring” effect on the SAR profiles, which reduces local gradients in SAR, thereby losing relevant information about the heating and focusing characteristics. The SAR is related to the initial temperature rise as

2 $$SAR=c\frac{\Updelta T}{\Updelta t},$$

where *c* (J/kg/°C) is the specific heat capacity, *∆T* (°C) the temperature rise, and *∆t* (s) the time of the power pulse. Regular QA measurements ensure good system performance and reliable phase-amplitude steering during treatments.

In clinical hyperthermia, steering strategies were traditionally mainly empirical, based on a protocol and the experience of the operator or hyperthermia center. Hyperthermia treatment planning in locoregional hyperthermia applies computer simulations to provide valuable insights into the effects of different steering strategies on the SAR/temperature distribution in the patient in terms of combining effective tumor heating and normal tissue hotspot suppression. Over the past decade, treatment planning has been increasingly used in the clinical workflow, assisting the operator to ensure treatment quality [20–22]. The supportive use of planning is also recommended in QA guidelines [16].

Several commercial software packages are commonly used for hyperthermia simulations. Examples of general simulation packages are Sim4Life (SPEAG, Zurich, Switzerland), COMSOL Multiphysics (Palo Alto, CA, USA), Ansys High-Frequency Structural Simulator (HFSS; Canonsburg, Pennsylvania, USA), and CST MW Studio (CST MWS; Computer Simulation Technology, Darmstadt, Germany). Sigma HyperPlan (Dr. Sennewald Medizintechnik GmbH) is a dedicated treatment planning package for use in combination with the locoregional BSD-2000 hyperthermia systems [23, 24]. Plan2Heat is a versatile planning package developed at Amsterdam UMC, supporting treatment planning for a wide variety of heating devices [25] including the locoregional BSD-2000 and ALBA-4D systems [26], superficial applicators [27, 28], and different capacitive devices [29–31]. Plan2Heat has been commercialized by Med-Logix srl (Rome, Italy) and is in use in several centers in combination with ALBA devices for superficial and deep heating.

When performing treatment planning for phased-array systems to assist in phase-amplitude steering, the basic heating characteristics as observed in QA measurements should be adequately predicted, i.e., within the range of measurement uncertainties (typically ~10–20% [32, 33]). This requires validation of the 3D model implementation of the heating device with comparison of the simulated and measured behavior of SAR patterns. Although applications of treatment planning are rapidly increasing, such planning validation studies using phantom measurements are relatively sparse and have used academic, in-house-developed, software. Studies in the 90s focused on validation of in-house-developed finite difference time domain (FDTD) software [34–37] and finite element software [37, 38] for BSD-2000 devices. Wiersma et al. validated the implementation of the AMC‑4 system in their in-house-developed software using the conjugate-gradient FFT method [32]. These validation studies used academic software that is no longer in use and considered only one specific phased-array heating device.

As mentioned above, Plan2Heat is a versatile planning software package suitable for modelling a wide variety of heating devices from multiple vendors. So far, studies validating planning predictions in phantom set-ups using modern treatment planning software are lacking in the literature. The purpose of this study was to evaluate the validity of model implementations of various phased-array systems in Plan2Heat. This validation will consider four different phased-array systems that are all used in daily clinical practice, i.e., the AMC‑2 and the AMC-4/ALBA-4D waveguide systems and the BSD Sigma-30 and Sigma-60 devices. Measurements in tissue-equivalent phantoms for these devices have been reported in the literature and these data will be used for validation.

## Methods

Four different phased-array devices were considered in this study: the AMC‑2, the AMC-4/ALBA-4D, and the BSD Sigma-30 and Sigma-60 devices, all of which are used in routine clinical practice. The AMC‑2 system and the AMC-4/ALBA-4D system are both waveguide systems, and the Sigma-30 and Sigma-60 are dipole systems.

3D models of these devices were implemented in the hyperthermia treatment planning software Plan2Heat. Plan2Heat is a versatile and flexible module-based treatment planning package containing modules for electric field and temperature calculations for various heating techniques, as well as several optimization routines and plan evaluation tools. The electromagnetic solver of Plan2Heat uses voxel-based GPU-accelerated FDTD calculations [25] with perfectly matched layer (PML) boundary conditions. The user can specify electromagnetic and thermal properties for different structures and tissues as appropriate for the specific simulations performed. The resolution applied for all cases reported in this paper was 2.5 × 2.5 × 2.5 mm^3^, which is similar to the resolution applied in treatment planning for routine clinical use at our institute [21, 39]. The models of the heating devices and phantoms were constructed by combining 3D geometrical objects (e.g., block, cylinder, ellipse). Waveguide sources for the AMC‑2 and AMC-4/ALBA-4D devices were positioned between the choke and the wall [40]. The Sigma-30 and Sigma-60 devices have flat copper dipole antennas, which were approximated as cylindrical dipoles [34, 41]. Dipole pairs were modelled by exciting two dipoles simultaneously, with the sources positioned in the gap between two dipole arms [42]. Connectors between the dipoles were thus not modelled explicitly. Sources were excited by means of a triple cosine pulse, which yields a shorter excitation time compared to a Gaussian pulse, thus enabling faster convergence (with minor differences) [43]. A total of 16 PMLs were used, and E‑fields were calculated for each individual antenna (pair) with unit amplitude and zero phase. Non-radiating antennas or antenna pairs were terminated by the theoretical characteristic impedance of 50 Ω. The total E‑field for specific phase-amplitude settings was calculated by superposition, and the SAR follows from Eq. 1. For evaluation, 1 cc-averaged SAR values were calculated.

All measurements used in this study to validate our model implementations were taken from literature reports based on either E‑field or ∆T measurements. An overview of the studies used is listed in Table 1, also summarizing phantom characteristics and the phase-amplitude settings applied. Some studies had to be disregarded, as summarized in supplementary Table S1. The studies included typically reported plots of the E‑field (profiles), ∆T (points), or SAR based on these measurements, which we digitized (https://www.digitizeit.xyz/) and converted to (normalized) SAR values using Eqs. 1 and 2 if needed. In the remainder of the paper, SAR profiles based on these measurements are referred to as “measured SAR profiles.”

**Table 1:** Overview of the studies used in this study to validate our model implementations of phased-array devices in Plan2Heat, also summarizing phantom characteristics and phase-amplitude settings applied

	Type of measurement	Published data	Phantom	Frequency	σ (S/m)	ε (−)	Power ratio	Phase
**AMC‑2**	**E‑field**	**Normalized E‑field **[44]	**Homogeneous**	**70** **MHz**	**0.51**	**77**		
*Settings:*								
Top							1	0°
[fixed]								
Bottom							1	−120°
Bottom							1	−90°
Bottom							1	−45°
Bottom							1	0°
Bottom							1	45°
Bottom							1	90°
Bottom							1	120°
**AMC-4/ALBA-4D**	**E‑field**	**E‑field [a.u]** [33]	**Homogeneous**	**70** **MHz**	**0.55**	**75**		
*Settings:*								
Top							1	0°
Bottom							1	0°
Left							1	25°
Right							1	25°
**AMC-4/ALBA-4D**	**∆T**	**SAR **[45]	**Inhomogeneous (central fat tube)**	**70** **MHz**	**0.55/0.035**	**75/18**		
*Settings:*								
Top							1	0°
Bottom							1	0°
Left							1	45°
Right							1	45°
Top							5	0°
Bottom							5	0°
Left							1	0°
Right							1	0°
**Sigma-30**	**∆T**	**Normalized SAR **[41]	**Homogeneous**	**130** **MHz**	**0.7**	**80**		
*Settings:*								
Top							1	0°
Bottom							1	0°
Left							1	0°
Right							1	0°
**Sigma-60**	**∆T**	**∆T/min **[34]	**Homogeneous with fat layer**	**70–110** **MHz**	**0.68/0.05**	**70/8**	^**a**^	^**a**^

**Fig. 1:**
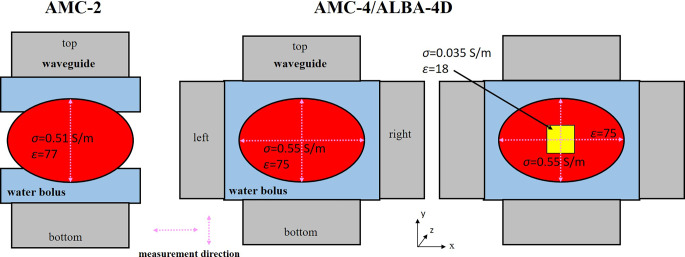
Schematic representation of the phantom measurement set-ups for the AMC-2 and AMC-4/ALBA-4D (Med-Logix srl, Rome, Italy) systems used for validation of treatment planning; σ conductivity (S m^−1^) and ε permittivity. Measurements were taken from Schneider et al. and Van Stam et al. [33, 44, 45]. The *pink horizontal *and *vertical dotted lines *indicate the measurement direction. Measurements were taken in the central transverse midplane and, for the inhomogeneous phantom, also 10 cm from the central midplane (z = 10 cm). The settings used are indicated in Table 1

**Fig. 2:**
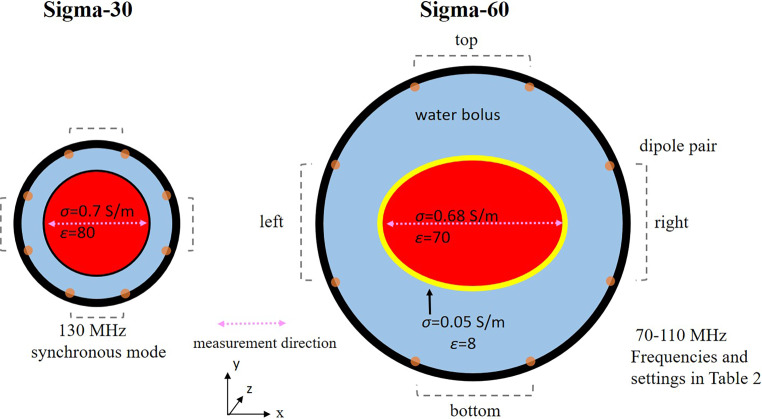
Schematic representation of the phantom measurement set-ups for the BSD Sigma-30 and Sigma-60 devices (Pyrexar Medical, Salt Lake City, UT, USA) used for validation of treatment planning; σ conductivity (S m^−1^) and ε permittivity. Measurements for the Sigma-30 and Sigma-60 were taken from Nadobny et al. [41] and Sullivan [34], respectively. Measurements were considered in the central transverse midplane and, for the Sigma-60, also 10 cm from the central midplane (z = 10 cm)

Top, bottom, left, and right refer to the top, bottom, left, and right waveguide or dipole pair (Figs. 1 and 2)

Amplitude settings are typically expressed as power ratios, where power is proportional to the amplitude squared

σ conductivity (S m^−1^), ε permittivity, *a.u*. arbitrary units

^a^Settings for the Sigma-60 are listed in Table 2

**Table 2:** Operating frequencies and power settings as reported in Sullivan [34] and used for validation of the simulations

	Focal point [x,y] (cm)		Top	Bottom	Left	Right
*70* *MHz*	[0,0]	Power ratio	1	1	1	1
		Phase^a^	0°	0°	0°	0°
	[−2,0]	Power ratio	0.72	0.72	1	0.47
		Phase^a^	−14°	−14°	−28°	0°
*80* *MHz*	[0,0]	Power ratio	1	1	1	1
		Phase^a^	0°	0°	0°	0°
	[−2,0]	Power ratio	0.74	0.74	1	0.5
		Phase^a^	−15°	−15°	−32°	0°
*90* *MHz*	[0,0]	Power ratio	1	1	1	1
		Phase^a^	0°	0°	0°	0°
	[2,0]	Power ratio	0.8	0.8	0.64	1
		Phase^a^	−17°	−17°	0°	−36°
	[4,0]	Power ratio	0.66	0.66	0.36	1
		Phase^a^	−34°	−34°	0°	−72°
*110* *MHz*	[0,0]	Power ratio	1	1	1	1
		Phase^a^	0°	0°	0°	0°
	[−2,0]	Power ratio	0.78	0.78	1	0.58
		Phase^a^	−21°	−21°	−44°	0°

Top, bottom, left, and right refer to the power ratio of the specific antenna pair (Fig. 2)

Amplitude settings are typically expressed as power ratios, where power is proportional to the amplitude squared

^a^The BSD control software automatically determines the phases settings based on the indicated focal point. Phases were not reported explicitly by Sullivan but were reconstructed for simulations using the distances between the antenna pairs and the focal point according to Eq. 3.

### Waveguide phased-array systems

#### The AMC-2 system

The 70 MHz AMC‑2 system is an in-house-developed and in-house-built double-waveguide system for treating breast tumors exceeding 4 cm depth and semi-deep supraclavicular tumor locations [44]. The bottom waveguide aperture measures 20 × 34 cm and the waveguide is mounted in a revolving circular section of the table, such that it can be rotated over −180° to +180°. These waveguides are filled with deionized water and the dimensions of the bottom waveguide are the same as those used in the AMC-4/ALBA-4D system. For the top waveguide, three different aperture sizes are available: 20 × 34 cm, 15 × 34 cm, and 8.5 × 34 cm. The depth of the waveguides is 12 cm (i.e., ~ 1/4 λ). Phases and amplitudes can be adjusted for both waveguides separately.

E‑field measurements for a homogeneous phantom setup as reported by Van Stam et al. were considered (Table 1; [44]). A set-up with two 20 × 34 cm waveguides was considered, as this is the most commonly used clinical set-up and also provides an initial validation of the AMC-4/ALBA-4D system, as it corresponds to a set-up with only the top and bottom waveguides activated. This set-up is schematically shown in Fig. 1. Water boluses were 6 cm thick (i.e., minimal thickness at the center of the waveguide) and measured 32 × 40 cm and 36 × 40 cm for the top and bottom bolus, respectively. The 115 cm long phantom had an elliptical cross section of 24 × 36 cm and a 2 mm thick PVC shell. The phantom was filled with a muscle-equivalent 3 g/L saline solution, with conductivity σ = 0.51 S/m and permittivity ε = 77 at 70 MHz, assuming a water temperature of 22 °C. Equal amplitudes were used for both waveguides. The top waveguide was used as a reference (phase 0°) and the phase of the bottom waveguide was varied over a large range to evaluate the effect of phase steering: −120°, −90°, −45°, 0, +45°, +90°, and +120° [44].

Measured normalized E‑field profiles along the minor axis, as reported by Van Stam et al. [44], were converted to SAR profiles using Eq. 1. The SAR profile achieved with a bottom phase of 0° was used to normalize at 100% at the center of the phantom. All other profiles were scaled accordingly and compared with the simulated normalized SAR along the minor axis of the phantom.

#### The AMC-4/ALBA-4D system

The 70 MHz AMC‑4 system is an in-house-developed and in-house-built four-waveguide locoregional heating system, which has recently been commercialized as the ALBA-4D system (Med-Logix srl Rome, Italy). The waveguides are organized in a ring (i.e., top, bottom, left, and right) and the waveguide aperture is 20 × 34 cm. Phases and amplitudes can be individually adjusted. The water bolus covers a length of 40 cm in the axial direction. Measurements in both homogeneous and inhomogeneous phantoms as reported by Schneider et al. and Van Stam et al. were used (Table 1; [33, 45]). Both experimental set-ups are schematically shown in Fig. 1.

Schneider et al. reported E‑field measurements for a homogenous phantom set-up [33]. The 115 cm long elliptical phantom had a cross section of 24 × 36 cm and a 2 mm thick PVC shell. The phantom was filled with a 3 g/L saline solution at a temperature of 25 °C, with conductivity σ = 0.55 S/m and permittivity ε = 75 at 70 MHz. Equal amplitudes were applied for all waveguides, and the phases of the top and bottom waveguide were 0°; those of the left and right waveguide were 25°. Water bolus thickness was ~4 cm. E‑field profiles were measured along the minor and major central transverse axes of the phantom and reported in arbitrary units. Reported profiles were converted to normalized SAR and compared with simulations.

Van Stam et al. reported absolute SAR measurements based on temperature rise (∆T) measurements in an inhomogeneous phantom set-up [45]. Here, a solid elliptical phantom measuring 50 cm was used with the same cross section as the homogeneous phantom. The phantom was filled with wallpaper paste consisting of 3 g/L NaCl and 26.8 g/L methylcellulose dissolved in water. This resulted in similar dielectric properties as for the homogeneous phantom (conductivity σ = 0.55 S/m and permittivity ε = 75). An inhomogeneity was realized by an 8 × 8 × 50 cm rectangular tube. This tube was filled with a fat-equivalent material consisting of 90% butanol, 10% water, 100 g/L carbomerum, and 10 g/L NaCl [46]. This resulted in a conductivity of 0.035 S/m and a permittivity of 18 at 70 MHz. The specific heat capacity was 4180 and 2430 J/kg/°C for the muscle-equivalent wallpaper paste and fat-equivalent material, respectively. Catheters with multi-sensor thermocouple probes were positioned in the transverse midplane and in the transverse plane 10 cm from the midplane, registering temperatures along the major and minor axes. Temperature rise measurements after ∆t = 100s were performed for two different phase-amplitude settings. First, all amplitudes were equal with a total power of 1000 W, and a phase shift of 45° was applied to the lateral waveguides relative to the top and bottom waveguide. In a second measurement all phases were equal, and the top and bottom waveguide delivered 300 W while the left and right waveguide delivered only 60 W. Reported measured SAR profiles were compared to simulations.

### Dipole phased-array systems

#### The BSD Sigma-30 system

The BSD Sigma-30 system is an annular phased-array system and is the smallest of the Sigma family. It is used for pediatric applications and tumors in extremities. The device consists of eight paired dipoles of ~23 cm in length, operating at a frequency in the range of ~115 to 135 MHz. Phases and amplitudes can be adjusted per individual dipole pair. The “30” refers to the diameter of the ring on which the dipoles are mounted, which is approximately 30 cm; the length is about 25 cm. The region between the antennas and the patient is filled up by a water bolus.

To validate our model implementation of the Sigma-30 in Plan2Heat, we used the measurements reported by Nadobny et al. [41] in a cylindrical agarose phantom 20 cm in diameter and 30 cm in length. This set-up is schematically shown in Fig. 2. The phantom material consisted of 4% agarose, ~3.7% sugar, and ~0.35% NaCl, thus yielding a conductivity of σ ≈ 0.7 S/m and a permittivity of ε ≈ 80 at approximately 25 °C and 125 MHz. The radial SAR profile with equal amplitudes and phases at 130 MHz was derived from temperature rise measurements using 250 W of power for 5 min. Temperature rises were measured using thermometry probes with a 1.5-cm spatial measurement resolution and a normalized SAR profile was reported, such that the relative SAR in the center of the phantom was 100% [41]. We compared this normalized profile to our simulated normalized SAR.

#### The BSD Sigma-60 system

The BSD Sigma-60 device is the most commonly used phased-array system of the Sigma family. This device also has eight paired dipole antennas (length ~45 cm), here mounted on a ring with a diameter of almost 60 cm (~58 cm) and a length of 50 cm. Phases and amplitudes can be adjusted per individual dipole pair. The water bolus fills the space between the patient and the antennas. The frequency range is variable between 60 and 120 MHz [5].

Temperature rise measurements from Sullivan were used, which were reported as ∆T/min [34]. In these early days, the water bolus was somewhat shorter (~36 cm) than in present-day Sigma-60 systems [47]. The experimental set-up used by Sullivan considered an elliptical CDRH (Center for Devices and Radiological Health) phantom with major and minor axes of 35 and 25 cm, respectively ([34, 48]; Fig. 2). The phantom consists of homogeneous muscle-equivalent material (σ = 0.68 S/m, ε = 70; 70–110 MHz) with a fat-equivalent layer 2 cm thick (σ = 0.05 S/m, ε = 8; 70–110 MHz). Thermometry catheters were positioned at 2‑cm intervals along the major and minor axes to determine the temperature rise per minute using a total 1200 W of power. Measurements were performed at the central transverse plane as well as 5, 10, and 15 cm from the central transverse plane. Frequencies varied between 70 and 110 MHz, with different phase-amplitude settings to realize power steering along the major axis. Evaluated frequencies and settings are listed in Table 2.

Given the focal point location, phases *φ*_*i*_ (°) for antenna pair *i* can be calculated using physical distances according to [34]:

3 $$\varphi _{i}=0.1\cdot f\cdot \left(D_{i}-D_{\max }\right),$$

where *f* is the operating frequency in MHz, *D*_*i*_ the distance (in centimeters) from the focal point to the antenna pair (quadrant) *i*, and *D*_max_ is the distance to the furthest quadrant, which will then serve as the reference antenna pair (i.e., phase 0°). Temperature rise per minute yields absolute SAR measurements according to Eq. 2, provided that the exact value of the specific heat capacity is known. Farina et al. characterized thermal properties of three different commonly used hyperthermia phantoms and their measurements showed a relatively large variation in the value of *c *(3850 ± 450 J/kg/°C) [49]. The value of *c* for the CDRH phantom was not specified and this uncertainty range is too large to reliably quantify the SAR. Therefore, we converted the temperature rise measurements to normalized SAR (i.e., 100% at the center of the phantom). Measured and simulated profiles were compared along the central major axis as well as along the major axis 10 cm from the central transverse plane.

## Results

### Waveguide systems

#### The AMC-2 system

**Fig. 3:**
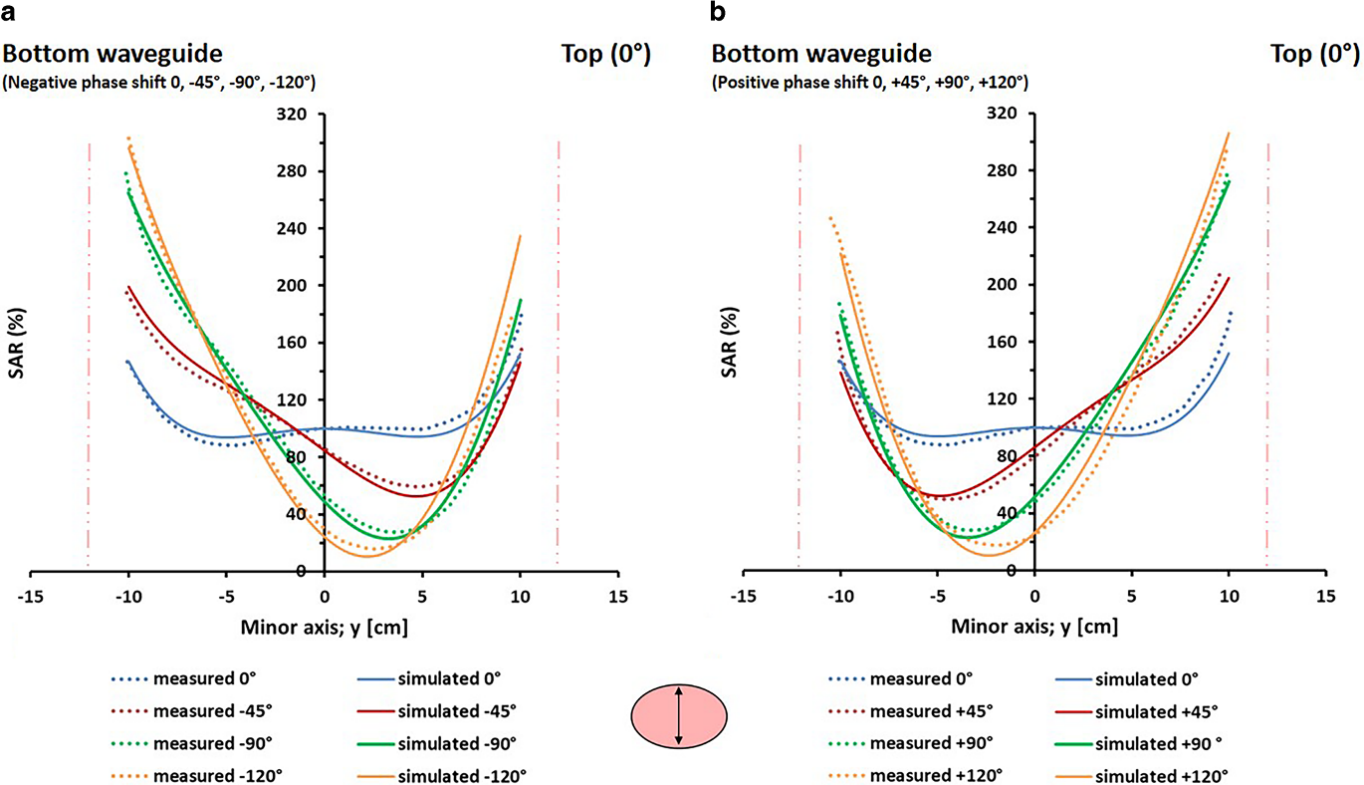
Measured and simulated specific absorption rate (SAR) profiles for a homogeneous elliptical phantom in the 70 MHz AMC‑2 system. Equal amplitudes were used and phase differences between the top and bottom waveguide varied between 0° and −120° (**a**) and between 0° and +120° (**b**). Measurements and simulations were normalized such that the central SAR value corresponds to 100% for equal phases. The *vertical lines* indicate the locations of the phantom borders. Measurements were taken from Van Stam et al. [44]

Figure 3 shows the measured and simulated SAR profiles for a homogeneous elliptical phantom heated with the AMC‑2 system using equal amplitudes and varying phase shifts between 0° and −120° (Fig. 3a) and between 0° and +120° (Fig. 3b) for the bottom waveguide. With equal phases (0°), a central SAR zone with a size of ~10–15 cm is observed in both measurements and simulations. An increasingly negative bottom phase shifts the SAR towards the bottom waveguide, with an increasingly more pronounced SAR minimum, which is almost zero near the center with a phase shift of −120° (yellow curve in Fig. 3a). For positive phase shifts the patterns are mirrored, as expected, moving the high SAR towards the top waveguide with increasing phase shift. Measured and simulated profiles show excellent agreement, with deviations < 10%. The results indicate that the effect of phase steering using these rectangular waveguides (as also applied in the AMC-4/ALBA-4D system), i.e., the shift of maximum and minimum SAR over the phantom depth, is well predicted by the simulations.

#### The AMC-4/ALBA-4D system

**Fig. 4:**
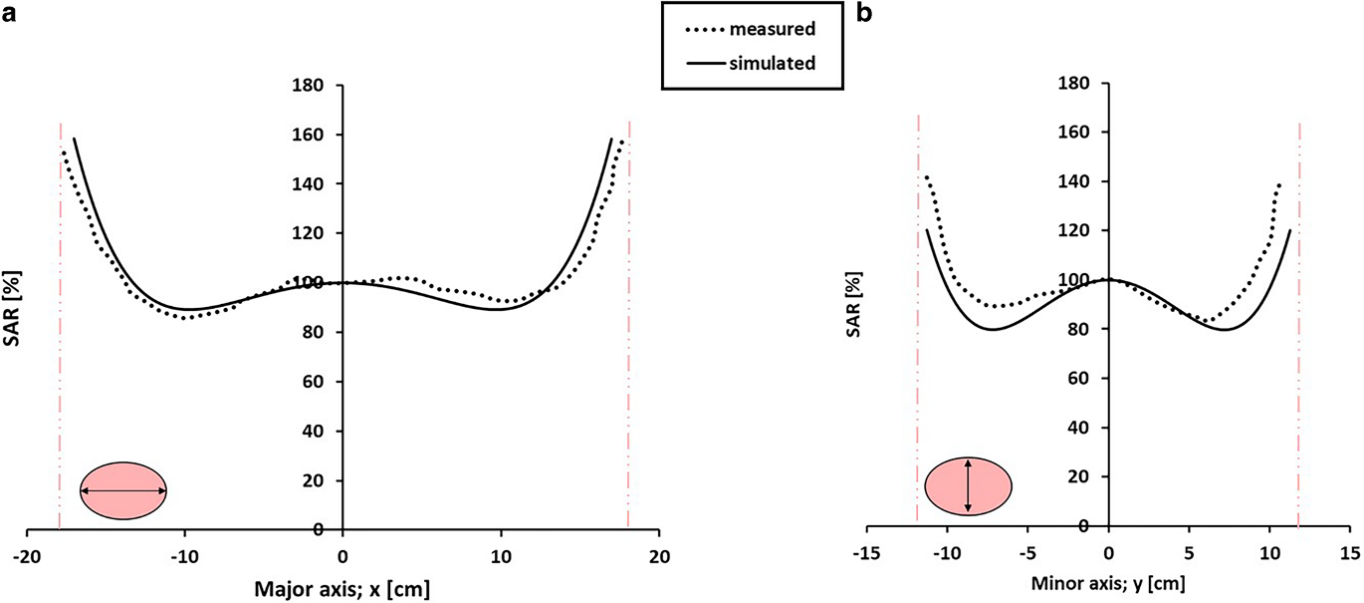
Measured and simulated SAR profiles along the major (**a**) and minor (**b**) axes for a homogeneous elliptical phantom in the 70 MHz AMC-4/ALBA-4D system. Equal amplitudes were used and phase settings top:bottom:left:right were 0:0:25:25 (°). Measurements and simulations were normalized such that the central SAR value corresponds to 100%. The *vertical lines* indicate the locations of the phantom borders. Measurements were taken from Schneider et al. [33]

Measured and simulated SAR profiles along the major and minor axes of a homogeneous phantom heated with the AMC-4/ALBA-4D system are shown in Fig. 4. Equal amplitudes were applied, and the left and right waveguide had a 25° phase shift with respect to the top and bottom waveguides. Simulations adequately predict the measured profiles within the range of the measurement accuracy (i.e., typically ~10–20% [32, 33]). The dimensions of the focus size, here considered as the distance between the two local minima in the profile, also correspond well. The size of the focal zone along the major axis is 20 cm, both measured and simulated. Along the minor axis the measured and simulated focus sizes are 13.5 and 14.5 cm, respectively.

**Fig. 5:**
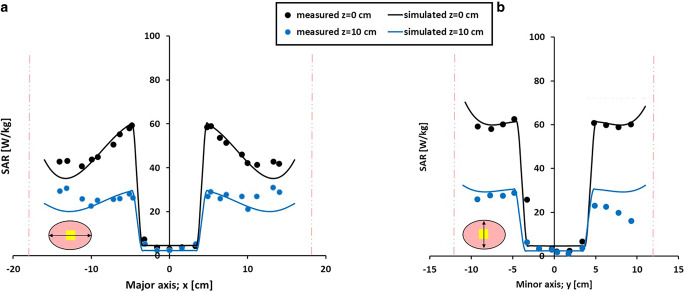
Measured and simulated SAR profiles along the major (**a**) and minor (**b**) axes for an inhomogeneous elliptical phantom in the 70 MHz AMC‑4 system. The muscle-equivalent phantom has a fat-equivalent central core. A total power of 1000 W was used with equal amplitudes; phase settings top:bottom:left:right were 0:0:45:45 (°). The *vertical lines* indicate the locations of the phantom borders. Measurements were taken from Van Stam et al. [45]

**Fig. 6:**
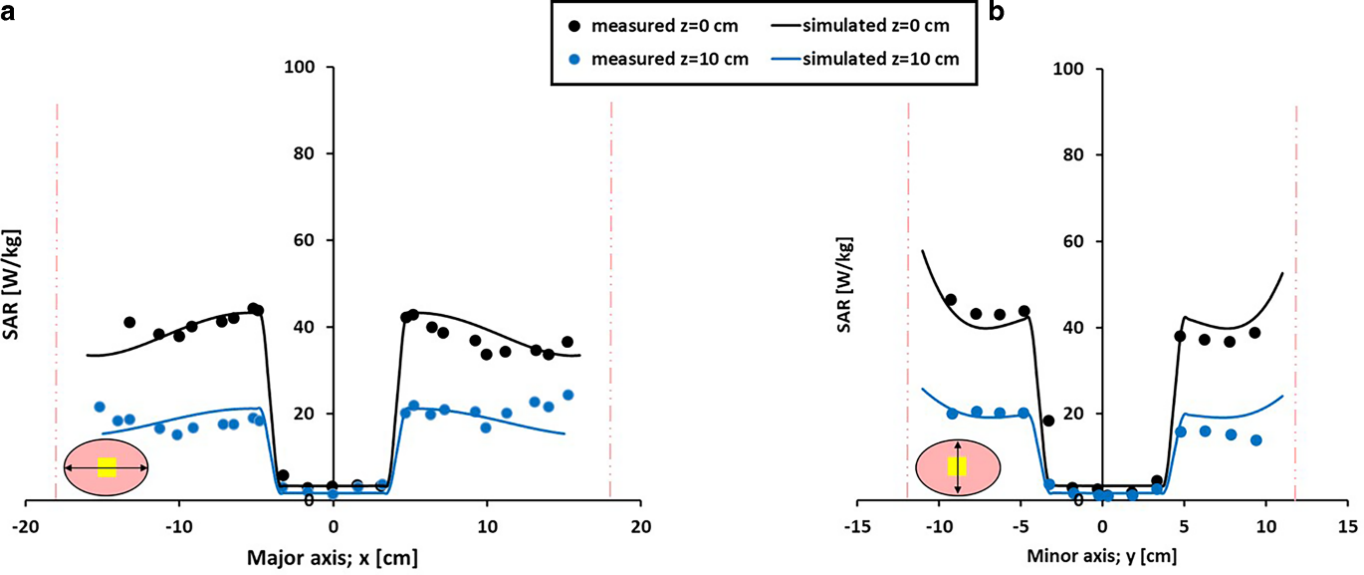
Measured and simulated SAR profiles along the major (**a**) and minor (**b**) axes for an inhomogeneous elliptical phantom in the 70 MHz AMC‑4 system. The muscle-equivalent phantom has a fat-equivalent central core. The top and bottom waveguides each delivered 300 W and the left and right waveguide 60 W, with equal phases (0°). The *vertical lines* indicate the locations of the phantom borders. Measurements were taken from Van Stam et al. [45]

Figures 5 and 6 compare measured and simulated SAR profiles along the major and minor axes for an inhomogeneous phantom with a fat-equivalent central core. Profiles were taken both through the central midplane and through the plane 10 cm from the midplane (z = 10 cm). In Fig. 5, all waveguides emitted the same amount of power, and the left and right waveguides had a 45° phase shift with respect to the top and bottom waveguides. In Fig. 6 all phases were equal, and the top and bottom antennas delivered five times as much power as the lateral antennas. This resulted in a lower SAR near the fat-equivalent core compared to the set-up with equal amplitudes, which is well predicted by the simulations. For both set-ups, at z = 10 cm, the SAR level is reduced by roughly 50%, both in measurements and in simulations, indicating an adequate prediction of the focus size. Overall, for the AMC-4/ALBA-4D system, a good match is observed between measurements and simulations within the range of the measurement accuracy (~10–20% [32, 33]). The effect of phase-amplitude steering on the SAR values is well predicted.

### Dipole systems

#### The BSD Sigma-30 system

**Fig. 7:**
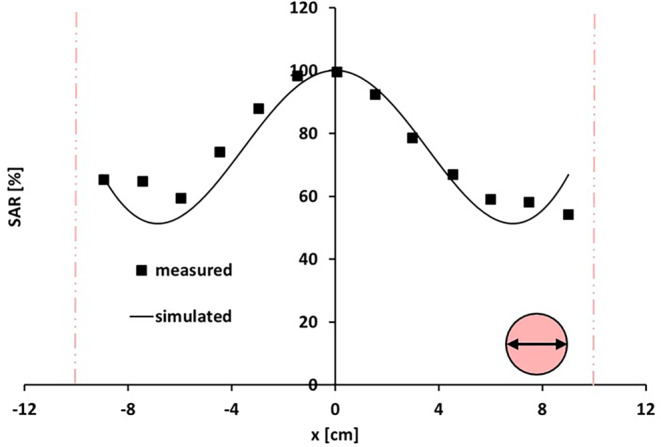
Measured and simulated SAR profiles along the left–right axis of the central transverse midplane of a homogeneous cylindrical phantom in the BSD Sigma-30 system at 130 MHz. Equal amplitudes and phases were used. The *vertical lines* indicate the locations of the phantom borders. Measurements and simulations were normalized such that the central SAR value corresponds to 100%. Measurements were taken from Nadobny et al. [41]

Figure 7 presents results of measurements and simulations of the normalized SAR profile along the horizontal axis of the transverse midplane in a homogeneous cylindrical phantom heated with the BSD Sigma-30 device operating at 130 MHz with equal amplitudes and phases. The agreement between measurement and simulation is very good, with deviations typically < 10% (i.e., within the range of the measurement accuracy). Along the SAR profile, a decay in normalized SAR from 100% to ~60% towards the left and the right sides is observed, which is well represented by the simulated profile, in terms of both relative decay as well as the position of the minimum (i.e., at approximately −7 cm and +7 cm).

#### The BSD Sigma-60 system

**Fig. 8:**
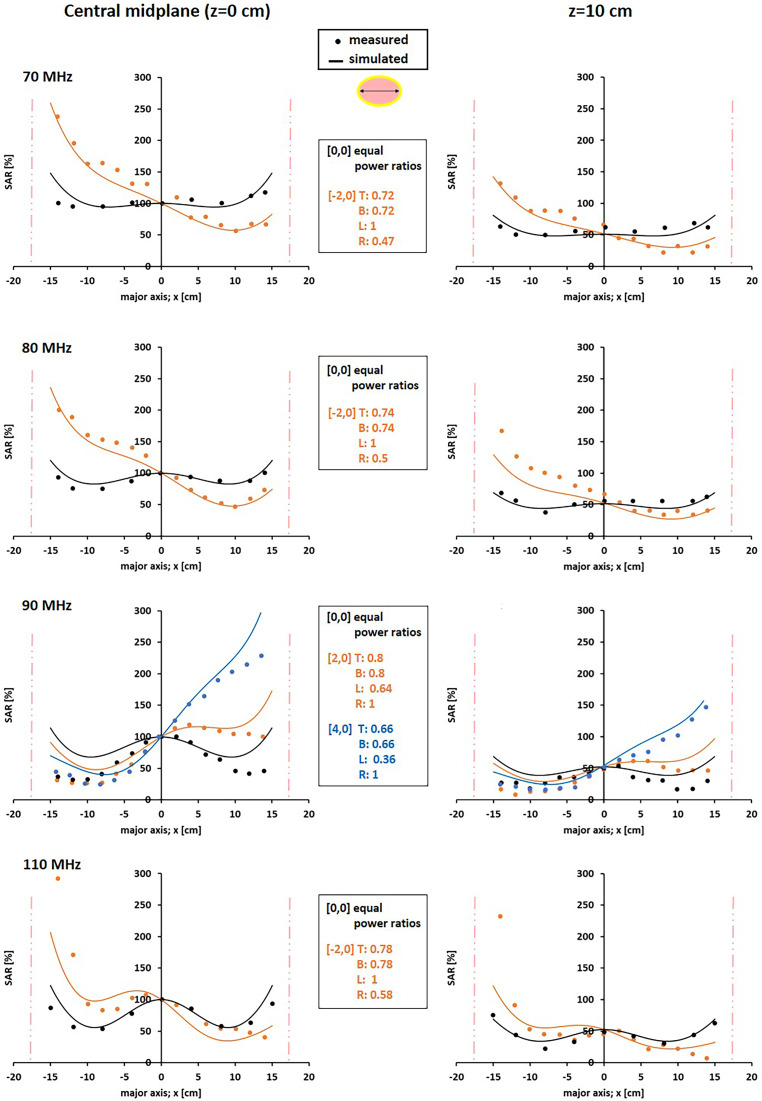
Measured and simulated normalized SAR profiles along the central major axis of the central transverse midplane (z = 0) and the major axis 10 cm from the central transverse axis (z = 10 cm) of a CDRH elliptical phantom in the BSD Sigma-60 system at frequencies of 70, 80, 90, and 110 MHz. Equal amplitudes and phases were used (*black profiles*) as well as different power ratios and focus points (*orange and blue profiles*). The values top:bottom:left:right (T:B:L:R) in the legends are the power ratios used in combination with the indicated focal point [x,y], as listed in Table 2. The *vertical lines* indicate the locations of the phantom borders. Measurements and simulations were normalized such that the central SAR value ([x,y,z] = [0,0,0]) corresponds to 100%. Measurements were taken from Sullivan [34]

Measurements and simulations of the normalized SAR in a homogeneous phantom surrounded by a fat-equivalent layer (CDRH phantom [48]) heated by the BSD Sigma-60 device are shown in Fig. 8. The measurement data were taken from Sullivan [34]. Different frequencies were evaluated, along with various focal points and power ratios as listed in Table 2. Figure 8 shows profiles taken along the major axis of the central transverse plane (z = 0 cm) and also the major axis profiles 10 cm from the central midplane (z = 10 cm). The size of the simulated focal zone along the major transverse axis is ~15–20 cm. With equal amplitudes and phases (focus position [0.0]), increasing the frequency yields a more pronounced central focal zone. These observations are in line with the measurement data. Selecting a focal zone at the left or right of the center produces a shift of the SAR profile, and the SAR shift in the measurements is well predicted by the simulations. Comparing the SAR levels in Fig. 8 for z = 0 cm and z = 10 cm also shows that at z = 10 cm, the SAR level is reduced by roughly 50% in both measurements and simulations. This indicates that the SAR decay over the longitudinal axis of the phantom, and thus also the axial focus size, is well predicted by the simulations. Overall, simulations are capable of predicting the basic heating characteristics of the Sigma-60, i.e., the approximate size of the focal zone and the effects of frequency and phase-amplitude steering on the SAR patterns.

## Discussion

This paper shows that the model implementation of four clinically used phased-array systems (AMC‑2, AMC-4/ALBA-4D, Sigma-30, and Sigma-60) in Plan2Heat allows for adequate prediction of the heating characteristics of these devices (i.e., focus size and effects of phase-amplitude and frequency steering), as observed in standard QA measurements in tissue-equivalent phantoms. Deviations between measurements and simulations were typically in the order of the measurement accuracy (i.e., typically ~10–20% [32, 33]). This range of measurement accuracy was observed in the reproduced experimental data, where most measured profiles show some asymmetry, although in theory they should be symmetrical. Experimental errors in this ~10–20% range are typical for QA measurements in locoregional hyperthermia. Wiersma et al. performed repeated E‑field scans in a homogeneous phantom and reported variations of more than 10% [32]. These experimental errors can be attributed to general set-up uncertainties, uncertainties in water bolus positioning, bolus contact with the phantom, phase and amplitude uncertainties, etc. In regions with relatively large gradients, e.g., near the phantom border, measurements become more sensitive to deviations. Furthermore, near the phantom borders, temperature rise measurements tend to be somewhat less reliable than E‑field measurements, since the measured temperature rise can be affected by influences of the nearby water bolus. Other factors influencing measurement accuracy are caused by differences in antenna matching [41] and crosstalk [50]. For the BSD Sigma-60 device, crosstalk effects typically tend to increase at higher frequencies [34, 36, 37, 50]. Capturing this phenomenon in simulations is challenging and requires adequate modelling of the impedance-matching networks [51].

Uncertainties in QA measurements of typically 10–20% are inevitable and inherent to locoregional hyperthermia using phased-array devices but do not limit their practical applicability for evaluating the basic heating characteristics of a phased-array applicator. Validation of the model implementation of phased-array devices for treatment planning should thus focus on adequate prediction of the basic heating characteristics of a specific device in terms of general heating patterns and changes due to phase-amplitude steering, rather than aiming for a very exact and pointwise quantified match between simulations and measurements. Results from this paper clearly indicate that the basic characteristics are predicted well and that deviations in SAR profiles are typically in the order of the measurement accuracy. Deviations might be slightly larger close to the phantom borders, which could be explained by influences of the water bolus and relatively large local SAR gradients. Superficial SAR values near the phantom border are thus more difficult to measure. However, exact SAR values are also less relevant at these locations, since strong bolus cooling is applied in clinical practice. The effectiveness of bolus cooling extends up to about 3 cm below the skin and prevents development of significant temperature increase at the skin. Taking all this into consideration, we can conclude that the heating characteristics of these different phased-array devices are well represented by their model implementation in Plan2Heat, which is an essential basis for clinical use of treatment planning using these devices.

Scientific literature reporting on reliably measured SAR patterns in tissue-equivalent phantoms generated by phased-array systems is relatively sparse. We selected data suitable for validation of our phased-array model implementations, but also some potentially useful data had to be disregarded, as summarized in supplementary Table S1. For example, for the Sigma-60 system, ∆T measurements for the inhomogeneous Utah phantom have been reported [35, 38]. The Utah phantom represents a generalized model of the pelvic region and consists of fat- and muscle-equivalent material shaped as a torso, two legs, and a central canal. Unfortunately, the production process of this phantom was quite challenging, and the phantom was not stable over time [38]. As a result, uncertainties in the exact location of the interfaces exist, and interfaces also became less sharply defined, as reported by the authors and clearly evident in CT images of the phantom [38]. This reduces the applicability of the Utah phantom for reliable validation studies. However, such inhomogeneous phantoms or more dedicated anthropomorphic phantoms [52] are not required to validate the implementation of the applicator model or for prediction of the basic heating characteristics of a device. These device characteristics and possible deviations in predictions would become even less clearly visible using advanced anthropomorphic phantoms instead of homogeneous phantoms. Such more advanced phantoms would mainly be useful for validating the model representation of more detailed geometries and their dielectric interfaces, i.e., mimicking a patient model. Dielectric interfaces yield large gradients in local SAR, and adequate prediction of these effects depends strongly on the modelling resolution, which was a serious issue of concern in the past when the resolution was still limited to 1–2 cm because of computer limitations. Dedicated techniques such as quasi-static zooming were required to realize adequate resolution to reliably model tissue interfaces [53]. Nowadays, the availability of strong computing power in the form of GPUs has resolved this issue [54].

The lack of reliable measurement data also meant that we were unable to include the BSD Sigma-Eye device in this validation study (see also Supplementary Table S1). Although some studies in the literature have reported temperature rise and E‑field measurements in phantoms for the Sigma-Eye system [47, 55, 56], these measurements showed significant inconsistencies and incompleteness, which means that a reliable ground truth benchmark for validation is lacking. Measurements by Mulder et al. used MR thermometry to determine the temperature rise for multiple SAR focal points to visualize effects of phase-amplitude steering in the Sigma-Eye [55]. MR thermometry applies smoothing to deal with noise and artefacts, and under optimal conditions the accuracy in phantoms is ~± 0.5 °C, whereas conventional probe thermometry has an accuracy of ~± 0.1–0.2 °C [18, 52]. This makes it difficult to use SAR patterns based on MR thermometry as a quantitative benchmark for validation. Considering the central focus set-up, the MR thermometry measurements showed a larger full-width-half-maximum compared to measurements reported by Fatehi et al. using a Schottky diode E‑field sheet [47]. Unfortunately, Fatehi et al. did not apply different phase settings. As a result, predicted effects of phase steering cannot be validated using these data. Furthermore, they did not report measured profiles along the minor axis. Profiles along both the major and minor axes are crucial for validation of a model implementation of the Sigma-Eye since, in contrast to the Sigma-30 and Sigma-60 devices, this device is not rotationally symmetrical. Furthermore, the MR thermometry measurements by Mulder et al. showed a very pronounced SAR focus, without any significant superficial temperature increase at the top and bottom of the phantom in the central midplane (minor axis). Taking into consideration the dimensions of the phantom and the Sigma-Eye and the wavelength at 100 MHz, a non-negligible temperature increase would be expected due to relatively high local SAR values. This is also suggested by simulations from de Jong et al. and E‑field measurements by Turner et al., which reported high superficial SAR levels over the minor axis [56, 57]. However, Turner et al. only reported measurements for longitudinal focus steering and not for focus steering in the transverse plane. Furthermore, the spatial resolution of the E‑field measurements by Turner et al. was relatively low and the image quality of the published results was suboptimal for validation of treatment planning. Future publications on QA will hopefully provide a more reliable characterization of the Sigma-Eye system and enable model validation of this device in Plan2Heat. This will also provide essential insight into the heating characteristics of this device and serve as a benchmark for clinical QA measurements.

Once the SAR distribution for a specific phased-array applicator has been calculated, the subsequent steps of the treatment planning process do not further consider the explicit 3D applicator model. The SAR distribution is used as input for a thermal simulation to predict the corresponding temperature distribution, and phase-amplitude optimization methods can optimize tumor heating while avoiding excessive normal tissue temperatures [58–62]. The thermal solver of Plan2Heat uses the Pennes’ bioheat equation [63] combined with a discrete vasculature model [25, 64], as validated by Raaymakers et al. [65]. Efficient optimization tools are also available in Plan2Heat [25], thereby allowing Plan2Heat to predict and optimize SAR and temperature distributions for several clinical applications of treatment planning.

In clinical applications of treatment planning, one should be aware that exact quantitative plan description and treatment delivery as in radiotherapy are not feasible, and this is also not the main purpose of hyperthermia treatment planning. In general, quantitative reliability of predictions of SAR and temperature levels in patients is limited due to significant uncertainties in dielectric tissue properties and blood perfusion [66–68]. Dielectric tissue properties may vary substantially for individual patients [69] and the hyperthermia-induced increase in blood flow depends on the local temperature as well as on other factors like the age and physical condition of the patient [70]. MR-based methods for reconstruction of patient-specific input parameters are still under development and are not yet sufficiently accurate for routine clinical use [71–73]. Despite these uncertainties, treatment planning can be very useful to ensure and improve treatment quality, as discussed in a practical guide demonstrating how to effectively apply treatment planning to optimize treatment strategies in daily clinical practice [20]. Treatment planning can be very helpful to qualitatively compare different treatment strategies in order to determine the optimal strategy for individual patients prior to the first treatment [20, 22, 74].

Phase-amplitude adjustments during treatment are traditionally based on experience and/or steering protocols, which renders treatment quality strongly dependent on the experience of the institute or operator. An increasingly important application of treatment planning is therefore in assisting to determine adaptive treatment strategies [75, 76]. The concept of adaptive treatment planning relies on the assumption that changes in local SAR or temperature levels induced by phase-amplitude steering correlate with changes predicted by the simulations, even if the absolute SAR and temperature levels are not accurately predicted [76, 77]. Adaptive hyperthermia treatment planning could thus help to assist in determining optimal steering strategies to reduce local hotspots, while maintaining adequate tumor heating, or even to further improve tumor heating. First clinical applications of adaptive treatment planning have demonstrated the feasibility [39, 76, 78], and wider clinical implementation would likely reduce variations in treatment quality.

It is clear that validated planning models capable of predicting the basic heating characteristics of the specific devices concerned are essential for effective and reliable use of hyperthermia treatment planning. As demonstrated in this work, this is enabled by Plan2Heat for the AMC‑2, AMC-4/ALBA-4D, Sigma-30, and Sigma-60 devices. Previously published studies have validated model implementations of different capacitive heating devices and superficial contact flexible microstrip applicators (CFMAs) in Plan2Heat [27, 28, 31]. This demonstrates the versatile use of Plan2Heat as a reliable (adaptive) hyperthermia treatment planning package for various applicators used in the clinical routine of superficial and deep hyperthermia treatments.

## Conclusion

Model implementations of four different clinically used phased-array devices (AMC‑2, AMC-4/ALBA-4D, Sigma-30, and Sigma-60) in the hyperthermia treatment planning package Plan2Heat enable adequate prediction of the basic heating characteristics of these devices, i.e., focus size and the effects of phase-amplitude and frequency steering. This predictive value is an essential prerequisite for hyperthermia treatment planning in clinical applications, where it can be supportive in pretreatment planning and for on-line adaptive strategies to realize good treatment quality while minimizing treatment limiting hotspots.

## Supplementary Information

Supplementary Table S1
