# Supplementary material for: Validation of the implementation of phased-array heating systems in Plan2Heat
Source: Strahlenther Onkol. 2024 Aug 14;201(2):135–50. doi: 10.1007/s00066-024-02264-0 (PMC11754364; doi:10.1007/s00066-024-02264-0)
Supplement: Supplementary file 1 — Supplementary Table S1 [file 66_2024_2264_MOESM1_ESM.docx]

**Supplementary Table S1**

| **Study** | **Device** |  | **Reason for exclusion** |
| --- | --- | --- | --- |
| **Wiersma et al [1]** | AMC-4 |  | Only measurements with one active antenna reported. |
|  |  |  |  |
| **Fatehi et al [2]** | Sigma-60 |  | Incomplete data, i.e. no profiles with phase steering; Significantly smaller focus size compared to[3];  E-field calibration not reported. |
|  |  |  |  |
| **Van Rhoon et al [4]** | Sigma-60 |  | Significantly smaller focus size compared to[3];  E-field calibration not reported. |
|  |  |  |  |
| **Sullivan et al [5]** | Sigma-60 |  | Phantom unstable (Utah phantom). |
|  |  |  |  |
| **Jia et al [6]** | Sigma-60 |  | Phantom unstable (Utah phantom). |
|  |  |  |  |
| **Wust et al [7]** | Sigma-60 |  | No profiles inside the phantom reported; Exact position of E-field probe inside bolus unknown. |
|  |  |  |  |
| **Fatehi et al [2]** | Sigma-Eye |  | Incomplete data, i.e. profiles with phase steering and along minor axis not reported; Significantly smaller focus size compared to[8]; E-field calibration not reported. |
|  |  |  |  |
| **Mulder et al [8]** | Sigma-Eye |  | Temperature rise measured with MR-thermometry, which is not a golden standard because of uncertainties. Significantly larger focus size compared to[2]. |
|  |  |  |  |
| **Wust et al [9]** | Sigma-Eye |  | Incomplete data, i.e. no profiles with phase steering.  problems with matching and phase calibration. |
|  |  |  |  |
| **Turner et al [10]** | Sigma-Eye |  | Incomplete data, i.e. only axial steering applied;  low measurement resolution. |

***Table S1:*** *Overview of the most relevant studies in the literature reporting QA measurement data in phantoms for phased array devices that we excluded from our study, including reasons for exclusion.*

[1] Wiersma J and van Dijk JDP, RF hyperthermia array modelling; validation by means of measured EM-field distributions. *Int J Hyperthermia.* 2001;17:63-81.

[2] Fatehi D and van Rhoon GC, SAR characteristics of the Sigma-60-Ellipse applicator. *Int J Hyperthermia.* 2008;24:347-56.

[3] Sullivan D, Mathematical-Methods for Treatment Planning in Deep Regional Hyperthermia. *Ieee Transactions on Microwave Theory and Techniques.* 1991;39:864-872.

[4] Van Rhoon GC, Van Der Heuvel DJ, Ameziane A, Rietveld PJ, Volenec K, and Van Der Zee J, Characterization of the SAR-distribution of the Sigma-60 applicator for regional hyperthermia using a Schottky diode sheet. *Int J Hyperthermia.* 2003;19:642-54.

[5] Sullivan DM, Buechler D, and Gibbs FA, Comparison of Measured and Simulated Data in an Annular Phased-Array Using an Inhomogeneous Phantom. *Ieee Transactions on Microwave Theory and Techniques.* 1992;40:600-604.

[6] Jia X, Paulsen KD, Buechler DN, Gibbs FA, Jr., and Meaney PM, Finite element simulation of Sigma 60 heating in the Utah phantom: computed and measured data compared. *Int.J.Hyperthermia.* 1994;10:755-774.

[7] Wust P, Berger J, Fähling H, Nadobny J, Gellermann J, Tilly W, Rau B, Petermann K, and Felix R, Scanning E-field sensor device for online measurements in annular phased-array systems. *International Journal of Radiation Oncology Biology Physics.* 1999;43:927-937.

[8] Mulder HT, Curto S, Paulides MM, Franckena M, and van Rhoon GC, Systematic quality assurance of the BSD2000-3D MR-compatible hyperthermia applicator performance using MR temperature imaging. *Int J Hyperthermia.* 2018;35:305-313.

[9] Wust P, Beck R, Berger J, Fahling H, Seebass M, Wlodarczyk W, Hoffmann W, and Nadobny J, Electric field distributions in a phased-array applicator with 12 channels: measurements and numerical simulations. *Med.Phys.* 2000;27:2565-2579.

[10] Turner PF, Schaefermeyer T, Latta M, Lauritzen R, and Toolson Sells D, 3D heating pattern steering using the sigma eye phased array applicator controlled by a modified BSD-2000. *Hyperthermic Oncology 1996, Volume II. Proceedings of the 7th International Congress on Hyperthermic Oncology, Roma, Italy.* 1996;pp. 446-448.
